# Epigenetic Modifications of MiRNAs in Osteoarthritis: A Systematic Review on Their Methylation Levels and Effects on Chondrocytes, Extracellular Matrix and Joint Inflammation

**DOI:** 10.3390/cells12141821

**Published:** 2023-07-11

**Authors:** Francesca Veronesi, Viviana Costa, Daniele Bellavia, Valentina Basoli, Gianluca Giavaresi

**Affiliations:** 1 Surgical Science and Technologies, IRCCS Istituto Ortopedico Rizzoli, Via Di Barbiano 1/10, 40136 Bologna, Italy; francesca.veronesi@ior.it (F.V.); daniele.bellavia@ior.it (D.B.); gianluca.giavaresi@ior.it (G.G.); 2 Department of Biomedical Engineering, Medical Additive Manufacturing Research Group (SwissMAM), University of Basel, 4123 Allschwil, Switzerland; valentina.basoli@unibas.ch; 3 Oral and Cranio-Maxillofacial Surgery, University Hospital Basel, 4031 Basel, Switzerland

**Keywords:** osteoarthritis, miRNA methylation, cartilage epigenetic modifications

## Abstract

Osteoarthritis (OA) is a joint disorder characterized by progressive degeneration of cartilage extracellular matrix (ECM), chondrocyte hypertrophy and apoptosis and inflammation. The current treatments mainly concern pain control and reduction of inflammation, but no therapeutic strategy has been identified as a disease-modifying treatment. Therefore, identifying specific biomarkers useful to prevent, treat or distinguish the stages of OA disease has become an immediate need of clinical practice. The role of microRNAs (miRNAs) in OA has been investigated in the last decade, and increasing evidence has emerged that the influence of the environment on gene expression through epigenetic processes contributes to the development, progression and aggressiveness of OA, in particular acting on the microenvironment modulations. The effects of epigenetic regulation, particularly different miRNA methylation during OA disease, were highlighted in the present systematic review. The evidence arising from this study of the literature conducted in three databases (PubMed, Scopus, Web of Science) suggested that miRNA methylation state already strongly impacts OA progression, driving chondrocytes and synoviocyte proliferation, apoptosis, inflammation and ECM deposition. However, the possibility of understanding the mechanism by which different epigenetic modifications of miRNA or pre-miRNA sequences drive the aggressiveness of OA could be the new focus of future investigations.

## 1. Introduction

Osteoarthritis (OA) is a joint disorder characterized by progressive degeneration of cartilage extracellular matrix (ECM), caused by the alteration between synthesis and degradation of its components such as collagen II (COL2A1) and Aggrecan, chondrocyte hypertrophy and apoptosis, subchondral bone sclerosis, osteophyte formation and inflammation [1,2]. Patients affected by OA experience high chronic pain, joint dysfunction and disability, temporary stiffness and swelling, leading to direct and indirect economic implications and social burdens [3]. Several risk factors have been identified, such as ageing, gender, injuries, obesity, genetic factors and abnormal mechanical loading [4]. It is estimated that nearly 40% of people over 70 are affected by OA, with a growing prevalence trend due to an ageing population [5,6]. Current treatments mainly concern pain control and reduction of inflammation. However, no therapeutic strategy has been identified as a disease-modifying treatment able to reverse the loss of cartilage ECM, and often joint replacement remains the treatment choice for the late stage of OA [7].

Regarding the pathophysiological process, it has been identified as a vicious circle in which cartilage degeneration and its products induce an increase in pro-inflammatory cytokines, the most abundant of which are interleukin 1β (IL1β) and tumour necrosis factor α (TNFα), which enhance the inflammatory response and, in turn, induce further degeneration and alterations of joint components (cartilage, synovium, bone), accelerating the OA process [8,9]. Some studies have elucidated the role of some molecular pathways (especially those regarding inflammation) implicated in the progression of OA [10]. However, the molecular therapy for OA disease is still under study. The possibility of using molecular targeted therapy would allow for the regulation of disease-related gene expression [11,12], which could help to slow down the progression of the OA disease.

Concerning this need, some studies identified microRNAs (miRNAs) as putative targets or vehicles of possible molecular therapy for tumour and OA diseases [13,14]. MiRNAs are small non-coding RNAs (nearly 22 nt) that are part of the miRNA-induced silencing complex (RISC) and are involved in the regulation or deregulation of gene expression in different pathophysiological processes [15]. They act by silencing the gene expression of targeted messenger RNA (mRNA) by binding to the 3′-untranslated region (3′-UTR) and inhibiting their translation or increasing their degradation [15,16], or acting as epigenetic regulators of gene expression [17].

In pathological situations like OA, miRNAs predominantly have a role in cartilage homeostasis and inflammation, modulating various cellular processes such as proliferation, apoptosis and differentiation [18]. Currently, 723 different miRNAs with differential expression between OA and healthy joint tissues have been identified [19,20], suggesting epigenetic regulation’s critical role in the disease’s progression [21,22].

Epigenetic regulation is another process that cells use to control the expression of a gene without changing the DNA sequence but instead influencing the way in which its sequence is read. Aberrant reading and controls during the epigenetic mechanism processes are correlated with the pathogenesis of various diseases [23]. In OA, the involvement of upregulated epigenetic mechanisms has been associated with the development and progression of disease [24]. In particular, the hypermethylation and hypomethylation in specific DNA CpG sites were localized in OA-related genes, inducing their silencing by inhibiting or limiting their binding to specific transcriptional factors or, in the case of hypomethylation, promoting the upregulation of a gene by increasing the region of binding [24,25]. Furthermore, role of miRNAs in the pathophysiology of OA has become more evident by having a pivotal role during the epigenetic controls, inducing the downregulation of their expression and upregulation of target genes [25].

The most critical actors playing a pivotal role in DNA methylation are a family of DNA methyltransferase (DNMTs), the most essential epigenetic regulators associated with bone or cartilage regeneration [26]. Indeed, it has been observed that the induction of cartilage ECM loss and chondrocyte hypertrophy are mediated by DNMT3B loss, underlying its role as an OA suppressor [27].

Given the ever-increasing importance of miRNAs in OA and the existing reviews on miRNAs in this pathology, this is the first review that evaluates miRNA methylation mechanisms in OA. The current systematic review collected and analysed in vitro studies that evaluated the epigenetic modifications, particularly methylation mechanisms of miRNA promoters and the modification of relative target genes involved in OA. Identifying a link between DNA methylation and miRNA expression during the disease progression could help understand how epigenetic mechanisms impact the diseases to develop a therapeutic approach against miRNA epigenetic modification.

## 2. Materials and Methods

### 2.1. Eligibility Criteria

In the present systematic review, we collect and include articles, considering parameters such as the population of interest (P), Intervention (I), Comparators and Outcomes (CO) formulating the PICO statement. The “Population” considered focused on the preclinical in vitro studies highlighting the role of different methylation states of miRNA promoters in OA disease. Studies that described the different methylation states of miRNA targets and the methylation of mRNA induced by miRNA regulation in OA disease were excluded. The “Intervention” item considered was any new technique used to identify the miRNA post-transcriptional modifications. The “Comparator” was any reference group of cells unaffected by OA (cells harvested from healthy donors).

The primary “Outcomes” were: (1) proliferation, (2) apoptosis and (3) the methylation state of miRNAs promoters. The secondary outcomes considered were (1) molecular investigations on miRNAs targets genes or (2) on protein releases. Reviews, editorials, commentaries, conference abstracts, studies published in a language other than English and articles without miRNA methylation descriptions were excluded, as well as clinical and in vivo studies.

### 2.2. Search Strategy

This systematic literature review involved a systematic search performed on 1 March 2023, conducted according to the Preferred Reporting Items for Systematic Reviews and Meta-Analyses (PRISMA) statement in three databases (www.pubmed.org, www.scopus.com, www.webofknowledge.com accessed on 1 March 2023). The keywords were: “(mirna methylation) AND (osteoarthritis)”. All in vitro studies without publication data limits (original articles in English and full text) were included. Two reviewers screened relevant articles using the title and abstract (F.V. and V.C.). The accepted articles were submitted to a public reference manager to eliminate duplicates and manage the references (Mendeley 1.14, www.mendeley.com). The included full-text articles were reviewed by the two authors, and any disagreement was resolved through discussion or with the involvement of a third author (D.B.). The following information from each paper was extracted and organized into Table 1 to summarize the evidence reported in each study: (a) aim; (b) in vitro model; (c) analyses; (d) results; (e) conclusions; and (f) references (Ref).

### 2.3. Risk of Bias Assessments within Individual Studies

There is not yet a validated tool to evaluate the internal validity of the included in vitro studies. Consequently, the internal quality of the collected references was defined by two different reviewers (F.V. and V.C.).

## 3. Results

**Figure 1:**
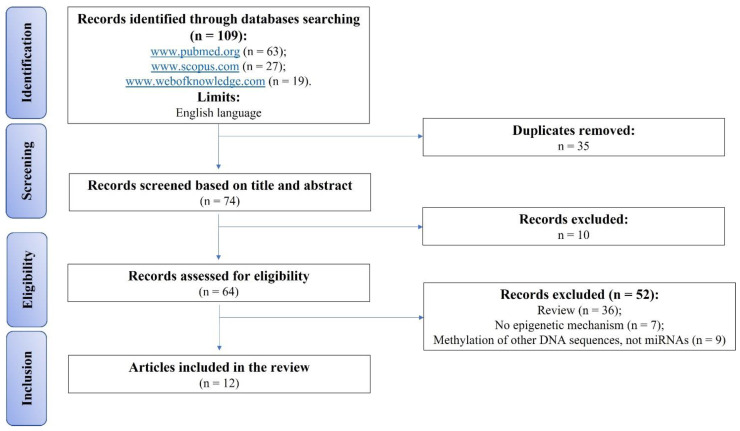
PRISMA flow diagram for the selection of studies.

An initial literature search retrieved 109 articles: 63 articles were identified using PubMed, 27 articles using Scopus and 19 were found in Web of Knowledge (Figure 1). Articles were submitted to a public reference manager (Mendeley 1.14, www.mendeley.com, accessed on 1 March 2023) to eliminate duplicates (n = 35). The remaining articles (n = 74) were evaluated by two independent researchers (F.V. and V.C.) by reviewing titles and abstracts. Subsequently, 64 complete articles were reviewed to establish whether the publication met the inclusion criteria, and 12 articles were recognized as eligible for this review and were included and analysed. Among the 52 full texts excluded, 36 were reviews, 7 concerned no epigenetic mechanism, and the other 9 were about the epigenetic mechanism of methylation but not of miRNAs (Figure 1). The 12 studies were grouped below based on miRNA upregulation or deregulation in OA tissues and cells (Table 1).

Several techniques were used for the analysis of the results. The authors of the collected studies employed various quantitative techniques: reverse transcription PCR (RT-qPCR) for the evaluation of gene expression [2,28,29,30,31,32,33,34,35,36,37,38]; western blot (WB) [2,28,31,32,33,34,35,36], immunoenzymatic assay (ELISA) [29,35], immunofluorescence (IHF) [31] or in situ hybridization [32] for the quantification of protein levels; MTT [31] or Cell Counting Kit-8 (CCK-8) [28,32,35,36] for cell proliferation assessment; and flow cytometry [28,29,30,31,32,33,34,35,38], TUNEL staining [33] or Annexin V assay [36] for cell apoptosis. In addition, methylation-specific PCR (MSP) [2,28,29,30,31,32,37,38] was employed for miRNA methylation analysis; dual-luciferase reporter gene assay [2,28,31,32,33,34,35,36] evaluated the direct relationship between miRNAs and their targets; chromatin immunoprecipitation (ChIP) [33,34,37] determined the specific locations in the genome associated with various histone modifications, indicating the target of the histone modifiers; and bisulphite sequencing [36,37] detected DNA methylation state. All of these techniques were performed in cartilage, chondrocytes or synovial fluid (SF) harvested from patients affected by OA [2,29,30,31,32,33,34,36,37,38], in purchased OA cell lines [2,35,37], or in rat or mouse chondrocytes cultured in the presence of IL-1β [28,33]. As a healthy control, the studies employed cells or cartilage from healthy donors, or cells cultured without inflammatory stimuli.

### 3.1. MiRNAs Upregulated in OA

Seven studies reported information on miRNAs modulation of three pathways related to cell proliferation/apoptosis [28,29,30,31,32,33], extracellular matrix (ECM) synthesis [2,32,33] and inflammation processes [32].

#### 3.1.1. Cell Proliferation/Apoptosis Pathway

Four out of seven studies observed the effect of miRNA methylation only in chondrocyte proliferation and apoptosis [28,29,30,31]. Yue et al. showed an increase of miR-574-5p promoter methylation and Bcl-2-associated X protein (Bax), a pro-apoptosis protein, and a decrease in cell proliferation in nuclear transcription factor YY1 Associated Factor 2 (*Yaf-2*) and anti-apoptotic protein B-cell lymphoma-2 (Bcl-2), while observing a negative correlation between miR-574-5p and *Yaf-2*, Bcl-2 and cell proliferation. MiR-574-5p acted by downregulating its target *Yaf-2*, increasing cell apoptosis and reducing cell proliferation. In addition, the authors observed that cryptotanshinone (CTS), a natural quinone compound, modulated the increase of miR-574-5p promoter methylation, thus reducing its expression [28]. In two other studies, Zhang et al. observed a reduction in small nucleolar RNA host gene 9 (SNHG9). In the circular RNA, circFADS2 correlates with increased CASPASE -3 (a lysosomal enzyme involved in the apoptotic pathway and consequently in cell apoptosis) [29,30]. The authors showed that overexpression of SNHG9 [29] and CircFADS2 [30] increased methylation of miR-34a [29] and miR-195-5p [30], resulting in a decrease in their expression. The study concluded that SNHG9 and CircFADS2 decreased chondrocyte apoptosis through miR-34a and miR-195-5p [29,30]. Dou et al. demonstrated a reduction in miR-39b promoter methylation, DNA methyltransferase 3 beta (DNMT3B), parathyroid hormone-like hormone (PTHLH) and cyclin-dependent kinase 4 (CDK4). DNMT3B induced hypermethylation of CpG sites in miR-39b promoter, which resulted in the downregulation of miR-39b expression in OA chondrocytes. Interestingly, the study described that PTHLH was a target of miR-39b, so its upregulation increased chondrocyte viability and suppressed their apoptosis through increased expression of CDK4 [31].

#### 3.1.2. Cell Proliferation/Apoptosis, ECM Synthesis and Inflammatory Pathways

Xiong et al. evaluated the epigenetic mechanism involving miRNAs in all three pathways: cell proliferation/apoptosis, ECM synthesis and inflammatory pathways [32]. They found an increase in metalloproteinases 3 and 13 (MMP3 and MMP13), which improved the degradation of ECM, inducible nitric oxide synthase (iNOS), cyclooxygenase 2 (COX2), TNFα and interleukin 6 (IL6), favoring inflammatory signalling and a decrease in COL2A1, myeloid cell leukemia-1 (MCL1; an apoptosis regulator and member of BCL-2 family protein), DNMT3B and miR-34a methylation. A positive correlation was found between miR-34a and pro-inflammatory factors, and a negative correlation between miR-34a and *COL2A1*, *MCL1* and *DNMT3B*. In particular, an inverse correlation between miR-34a methylation and DNMT3B expression with the direct interaction between MCL1 and miR-34a has been demonstrated, and the modulation of the phosphoinositide 3-kinase (PI3K)/protein kinase B (AKT) pathway involved in the control of intracellular signal transduction pathways that promotes metabolism, proliferation, cell survival and growth. Finally, the authors concluded that the *DNMT3B/miR-34a/MCL1* axis regulated chondrocyte viability, and DNMT3B/miR-34a/PI3K/AKT axis the ECM synthesis and inflammatory response [32].

#### 3.1.3. Cell Proliferation/Apoptosis and ECM Synthesis Pathways

Lian et al. demonstrated a reduction in autophagy-regulating protease 12 (ATG12), a positive mediator of mitochondrial apoptosis; microtubule-associated protein 1A/1B-light chain 3 (LC3), a central protein in the autophagy pathway where it functions in autophagy substrate selection and autophagosome biogenesis; EZH2, a transcriptional repressor; and the 27th amino acid in histone H3 (H3K27), a repressive transcriptional modifier. All of these proteins were negatively correlated with miR-128a overexpression. Induced overexpression of EZH2 in chondrocytes promoted methylation in H3K27 enrichment in the proximal region of miR-128a promoter, thus inhibiting miR-128a expression. MiR-128a, through the binding to 3′-UTR of *ATG12*, increased apoptosis (BAX, BCL-2, CLEAVED CASPASE-3) and reduced the chondrocyte ECM synthesis of SRY-box transcription factor 9 (SOX9), COL2A1 and AGGRECAN [33].

#### 3.1.4. ECM Synthesis Pathway

Kang et al. observed that low expression of small mother against decapentaplegic (SMAD3), a type of protein involved in the TGF-β signalling pathway involved in proliferation and differentiation, was correlated with miR-23a-3p promoter methylation. A negative correlation between *SMAD3* and miR-23a-3p was observed, suggesting that *SMAD3* is a target of miR-23a-3p and that the overexpression of miR-23a-3p reduced *COL2A1* and *AGGRECAN.* Therefore, the authors concluded that miR-23a-3p suppressed ECM synthesis through *SMAD3* regulation [2].

### 3.2. MiRNAs Downregulated in OA

Five studies evaluated the involvement of miRNAs in all the previously mentioned pathways [34,35,36,37,38], except for ECM synthesis.

#### 3.2.1. Cell Proliferation/Apoptosis and Inflammation Pathways

Three studies evaluated cell proliferation/apoptosis and inflammation pathways [34,35,36]. Wang et al. observed an increase in histone methylation of miR-138 promoter, cell apoptosis, MMP13, a disintegrin and metalloproteinase with thrombospondin motifs 4 and 5 (ADAMTS4 and *ADAMTS5*), two of the best matrix proteinases involved in the degradation of aggrecan, EZH2 and syndecan 1 (SDC1, an integral membrane protein involved in cell proliferation and migration). They found that these were negatively associated with miR-138 expression. Additionally, the silencing of EZH2 caused miR-138 promoter methylation to decrease while its expression increased. They observed that *SDC1* was a target of miR-138 and that the overexpression of both *EZH2* and miR-138 in chondrocytes reduced *SDC1*, apoptosis, *MMP13*, *ADAMTS4* and *ADAMTS5*, showing that EZH2 induced cartilage catabolism-related factors through miR-138/*SDC1* pathway [34].

Cai et al. found low cell viability and a strong correlation between fat mass and obesity-associated gene (FTO), Bcl-2 and miR-525-5p expression. High cell apoptosis and inflammation signalling were observed in an in vitro OA model of human cells. The authors revealed an increase of pro-apoptotic factors, such as BAX, CLEAVED-CASPASE-3 and pro-inflammatory soluble factors such as COX2, iNOS, IL6, IL1β and TNFα, as well as toll-like receptor 4 (TLR4), MYD88, P/T-P65 and P/T-IKBα expression and an up-regulation ofpri-miR-515-5p m6A methylation level. Overexpression of one of the fat mass and obesity-associated protein-alpha-ketoglutarate dependent dioxygenase (FTO) increased the binding affinity of pre-miR-515-5p to DGCR8 (a microprocessor complex which mediates the biogenesis of miRNAs from the primary miRNA transcript) and, consequently, miR-515-5p expression. Indeed, it was observed that FTO, interacting with DGCR8, modulated the processing of pre-miR-515-5p in an m6A-dependent manner. Finally, it was shown that miR-515-5p targeted *TLR4*, inhibiting the MyD88/NF-kB pathway and, thus, inflammation [35].

Cell viability and expression of miR-124 and miR-143 were reduced. In contrast, cell apoptosis, rho-associated, coiled-coil-containing protein kinase 1 (Rock1), nuclear factor kappa-light-chain-enhancer of activated B cells (Nf-kB) and Tlr9 were increased. These signalling pathways activate apoptosis by modulating the caspase protein pathway. OA chondrocytes cultured in the presence of exosomes derived from BMSCs and previously cultured with curcumin (CUR), a natural compound, reversed these results. It was observed that CUR increased miR-124 and miR-143 expression by reducing the methylation of their promoters. Finally, the authors demonstrated that miR-143 and miR-124 acted by inhibiting their gene targets, *Nf-kB* and *Rock1*, respectively, so reducing inflammation [36].

#### 3.2.2. Inflammation Pathway

Papathanasiou et al. demonstrated that chondrocytes and synoviocytes presented an increase in MMP13, ADAMTS5, interleukin-1 receptor-associated kinase 1 (IRAK-1), IL1β and IL6. Furthermore, chondrocytes showed high methylation of the miR-140-5p regulatory region, while synoviocytes showed methylation of miR-146a promoter, resulting in a decreased expression of miR-140-5p and miR-146a. Methylation impaired SMAD3 and NF-kB binding affinity on miR-140-5p and miR-146a regulatory regions, respectively. The authors concluded that methylation-mediated downregulation of miR-140-5p impacted *MMP-13* and *ADAMTS5* expression levels and that downregulation of miR-146a affected the expression of *IRAK-1*, *IL1β*, and *IL6* [37].

#### 3.2.3. Cell Proliferation/Apoptosis Pathway

Zuo et al. evaluated only the cell proliferation/apoptosis pathway. They observed an increase in a circular RNA derived from exons 12 and 13 of the HECW2 gene, the WW domain-containing E3 ubiquitin protein ligase 2 gene (Circ_HECW2). They found a negative correlation between Circ_HECW2 and miR-93. The overexpression of Circ_HECW2 induced miR-93 methylation, promoting the deregulation of miR-93 expression and an increase in apoptosis signaling activation. That suggests that Circ_HECW2 increases chondrocyte apoptosis through modifications of miR-93 methylation state [38].

## 4. Discussion

Several miRNAs have been identified as linked to OA progression, but their epigenetic modifications are still mainly under evaluation [39]. Epigenetic mechanisms, observed in many pathologies, are modifications in gene expression without changing the DNA sequence and depend on environmental factors [23]. Among these mechanisms, the DNA methylation of CpG sites is the most frequent [23]. MiRNAs themselves perform an epigenetic action on genes implicated in the pathogenesis of OA, and there is extensive literature describing their involvement [17]; however, few studies still deal with miRNA silencing due to DNA methylation.

This systematic review aimed to elucidate part of the molecular mechanisms involved in OA, providing more in-depth information on the molecular players by collecting and analysing in vitro literature studies that focused on epigenetic modifications and miRNA promoters, underlining the methylation on 13 miRNAs [2,28,29,30,31,32,33,34,35,36,37,38] that is known to be involved in the pathogenesis of OA [15]. These studies investigated the involvement of miRNA signaling in OA disease using gain and loss of function studies performed through infection or transfection approaches. To determine the post-transcriptional modification of mRNA, the elective method used was MeRIP-Seq. In OA models described in this systematic review, the effect of different miRNA methylation states on their specific targets (mRNA and relative proteins expression) was investigated using two different approaches: (i) a direct: dual luciferase assay or Chip methods and (ii) indirect: qRT-PCR, WB and ELISA assays.

The pleiotropic role of each single miRNAs in micro- and macro-environment in physiological and pathological conditions is known; however, this review highlighted which miRNAs are intensely involved in OA and act by modifying, directly or indirectly, three pathways: chondrocyte viability and apoptosis, synthesis of cartilage ECM components and inflammation, with most of them being involved in the first [28,29,30,31,32,33,34,35,36,38]. Indeed, cartilage ECM is continuously remodeled by the action of chondrocytes under the effect of inflammatory factors (MMPs) that are activated by inflammatory mediators, such as IL1β and TNFα, resulting in the degradation of COL2A1 and aggrecan, the most abundant proteins of ECM [40]. Chondrocytes, which are the only cells of cartilage, are responsible for the synthesis of ECM components, so their apoptosis is crucial in the degeneration of cartilage in OA [41]. Besides chondrocytes, synovium and synoviocytes play a pivotal role in the development of OA. Indeed, OA has been recognized as a degenerative and inflammatory disease that affects the whole joint, including cartilage, subchondral bone, synovium, muscles, menisci and ligaments [42]. Patients affected by OA demonstrate proliferation and inflammation of synoviocytes, synovitis and synovial lining hyperplasia. In OA, synoviocytes secrete inflammatory enzymes inducing cartilage destruction, thus aggravating synovial inflammation and forming a vicious cycle [43]. In the present review, four studies employed synoviocytes harvested from patients affected by OA [29,30,37,38].

**Figure 2:**
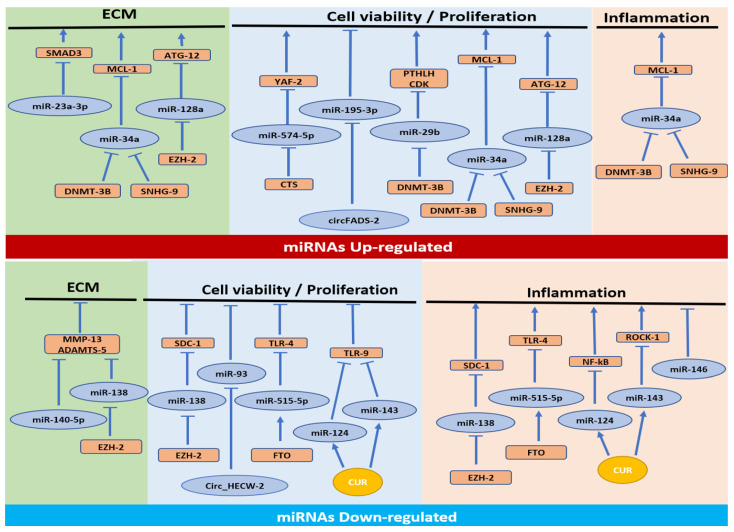
Epigenetic miRNA methylation and involved pathways in the OA pathology.

As shown in Figure 2, six out of thirteen miRNAs were found to be upregulated in OA tissues and cells (miR-574-5p, miR-34a, miR-195-3p, miR-29b, miR-128a and miR-23a-3p) [2,28,29,30,31,32,33]. Most of them are implicated in cell viability/apoptosis pathways since the upregulation of miR-574-5p [28], miR-34a [29,32], miR-195-3p [30], miR-29b [31] and miR-128a [33] reduces chondrocyte viability through the downregulation of their target genes YAF2 [28], PTHLH [31], CDK4 [31], MCL-1 [32] and ATG12 [33].

Some of the miRNAs mentioned above are also involved in the other two pathways of OA. MiR-34a is implicated in ECM synthesis and inflammation by downregulating MCL1 expression. MiR-128a is involved in reducing cartilage formation and cell viability response of chondrocytes regulating the expression of ATG12. The role of autophagy in OA development and progression is more investigated regarding the identification of modified genes or protein expression and relative miRNAs. However, evidence of the involvement of miRNAs promoter methylation in this phenomenon must be better understood [44]. Identifying a new target to prevent chondrocyte autophagy would allow the problems induced by the known treatment with caspase inhibitors which induce cancer-like effects to be overcome [44]. In addition, recent evidence suggests the protective role of autophagy during OA inflammatory response [45]. Among these miRNAs upregulated in OA, miR-23a-3p is involved only in the ECM synthesis pathway, downregulating SMAD3 and consequently the reduction of transforming growth factor (TGF)-β signaling. This signal plays a central role in maintaining articular cartilage [46] because it is involved in stimulating chondrocyte proliferation and inhibiting their terminal hypertrophic differentiation; the activation of SMAD3, through phosphorylation, is defined as protective against OA-related inflammation in chondrocytes [47]. These data are also confirmed by the study performed by Valdes A. et al. which demonstrated how a genetic variation in the SMAD3 gene is involved in genetic susceptibility to large-joint OA [48].

This review also highlights several different factors that induce the epigenetic methylation of these miRNAs, such as natural components [28], non-coding RNAs [29,30] and enzymes [31,32,33]. CTS, a water-soluble component of Salvia miltiorrhiza with anti-inflammatory and anti-tumour effects, can downregulate the expression of miR-574-5p by regulating its methylation, thus improving Yaf2 expression and affecting chondrocyte apoptosis [28]. However, Yaf-2 is also involved in the reduction of the anabolic metabolism of chondrocytes, leading to the decrease of COL2A1 and ACAN expression [49] and the suppression of chondrocyte inflammation [50] during OA progression. This evidence suggests a pleiotropic role of Yaf-2 in disease and its possible therapeutic role in OA treatment. SNHG9, a long non-coding RNA (>200 nt), which is involved in the regulation of other gene expression and interacts with Wnt2, is found to be downregulated in OA and can inhibit chondrocyte apoptosis by downregulating miR-34a through methylation [29].

CircFADS2, a covalently closed single-strand RNA that regulates gene expression in several pathologies, may downregulate miR-195-3p through methylation, suppressing chondrocyte apoptosis and OA [30]. DNMT3B is a DNA methyltransferase found to regulate cartilage homeostasis. It was evaluated in two studies involving two miRNAs, miR-29b and miR-34a [31,32]. Hypermethylation of specific CpG sites in the miR-29b and miR-34a promoter regions, induced by DNMT3B, contributes to the downregulation of miR-29b and miR-34a in OA chondrocytes. The silencing of miR-34a by DNMT3B could effectively reduce chondrocyte ECM degradation and inflammatory response by targeting MCL1 and mediating the downstream PI3K/AKT pathway. The epigenetic modulation on miR-29b promoter region, mediated by DNMT3, induces the upregulation of PTHLH expression and consequently (i) positive regulation of cartilage-specific marker expression (COL2A1, SOX9, ACAN) [51], (ii) enhancement of chondrocyte growth and (iii) suppression of their apoptosis and extracellular matrix degradation. Finally, the histone methyltransferase EZH2 is observed to be expressed at high levels in OA, and it induces methyl histone H3K27 enrichment in the miR-128a promoter, thus deregulating miR-128a transcription in inflamed chondrocytes [33].

A second group of seven miRNAs was found to be deregulated in OA: miR-138 [34], miR-515-5p [35], miR-124 [36], miR-143 [36], miR-93 [38], miR-140-5p [37] and miR-146a [37]. As shown in Figure 2, most of them are implicated in the inflammation pathway in OA pathology. The downregulation of miR-138 [34], mir-515-5p [35], miR-124 [36], miR-143 [36], miR-140-5p [37] and miR-146a [37] induces an increase in inflammatory molecules through the upregulation of their targets, SDC1 [34], TLR4, that inactivates MYD88/NF-kB pathway [35], Nf-kB [36,37], Tlr9 [36], Rock1 [36] and SMAD3 [37]. As also observed for the upregulated miRNAs, in this case, some of the miRNAs downregulated in OA are involved in other pathways: miR-138 [34], miR-515-5p [35], miR-124 [36] and miR-143 [36] are implicated in chondrocyte viability. Additionally, miR-93 is involved only in the chondrocyte viability pathway [38].

Some factors have been identified as targets that promote methylation or demethylation in these groups of miRNAs in OA, such as enzymes [34,35], natural components [36] and non-coding RNAs [38]. For example, EZH2, also observed for miR-128a expression, inhibits miR-138 expression by promoting the histone methylation of its promoter, which induces cartilage degeneration in OA models by upregulating SDC1 expression [34]. FTO, an m6A demethylase that mediates N6-methyladenosine demethylation, alleviates OA in an m6A-dependent manner via the miR-515-5p/TLR4/MyD88/NF-κB axis [35]. This signaling regulates cell viability and inflammation in disease progression; it was demonstrated as TLR4 e TLR9 is implicated in NF-kB activation and the pro-inflammatory phenotype of chondrocytes, leading to the hypertrophic condition [52]. Exosomes derived from CUR-treated MSCs significantly restore the down-regulated miR-143 and miR-124 expression in OA. CUR, a natural drug in wound healing with anti-cancer, anti-inflammatory, anti-oxidant and chemotherapeutic features, decreases the DNA methylation of miR-143 and miR-124 promoters, consequently attenuating OA [36]. miR143 down-regulation induces the inhibition of Rock-1 expression altering chondrocyte phenotype, improving: (i) the degradation of chondrocyte ECM; and (ii) the hypertrophy-like changes with the expression of various chondrocyte maturation leading to cartilage disruption and affecting OA pro-inflammatory pathological factors [53,54]. Circ_HECW2 and miR-93 were inversely correlated, with Circ_HECW2 upregulated and miR-93 deregulated in OA. Circ_HECW2 might inhibit miR-93 expression through methylation, thus affecting chondrocyte apoptosis [38].

The present review aimed to evaluate miRNA methylation modifications in vitro, under inflammatory stimuli, through which the epigenetic mechanisms of miRNAs and the factors contributing to these mechanisms. However, for a better understanding of the future possible interventional options, some in vivo studies are now briefly presented. Some included in vitro studies conducted their observations in OA in vivo models. They were performed in rodents in which OA was induced through monoiodoacetate (MIA) injection [28,35,36] and anterior cruciate ligament transaction (ACLT) + medial meniscus transaction (MMT) [32,34]. All of the in vivo results confirmed the in vitro findings previously discussed.

CTS downregulates miR-574-5p, which was found high in OA, through its methylation, thus reducing chondrocyte apoptosis [28]. Similarly, a high level of chondrocyte apoptosis was observed in OA mice, while the treatment with exosomes derived from CUR-treated MSCs suppressed chondrocyte apoptosis. Declined expression of miR-124 and miR-143 was observed in OA animals, while CUR treatment decreased the DNA methylation of miR-143 and miR-124 promoters, upregulating them and increasing chondrocyte proliferation and decreasing inflammation [36].

FTO and miR-515-5p expression were found to be low in OA rats. After injection of adenovirus containing FTO, miR-515-5p levels were up-regulated, the damage to cartilage structure was alleviated, and the Mankin score, apoptosis-related protein levels and inflammatory factor levels were reduced. These results indicate that overexpression of FTO increased miR-515-5p, favoring a less aggressive OA [35]. EZH2 was expressed at high levels in OA models and accelerated cartilage degeneration through methylation of miR-138 and downregulation of this miRNA [34]. Finally, DNMT3B silenced miR-34a, whose expression was high in OA. Up-regulation of DNMT3B reduced the expression of Mmp3, Mmp13, iNos, Cox-2, Tnfα and Il-6 and increased Col2a1 expression in cartilage tissues or synovial fluid of OA rats [32].

Another critical aspect of OA, which should have been considered in the studies included in this review, is the involvement of mechanotransduction, a term used to indicate molecular interactions that convert physical signals into cellular responses. Since OA is a whole-joint process, the mechanical load is considered a driver of the degenerative process [55]. However, the present review finds no miRNA promoter methylation changes under load. Given the importance of this aspect, future studies are needed to understand the mechanisms of epigenetic modifications better and learn to control them.

## 5. Conclusions

Understanding the role of epigenetic regulators in OA is crucial to harness the potential of epigenetic drugs. Here, we reviewed different hypothetical areas of opportunity for new possible targets for OA drugs that target epigenetic processes that involve miRNA regulation: cell proliferation, apoptosis and inflammation process.

We also discussed how understanding the interplay between OA epigenetics and miRNA target modification could provide the basis for novel treatment regimens combining epigenetic drugs and OA- inflammatory therapies.

Using proteins that can act by modifying the methylation profile of miRNA could help find innovative pharmacological therapies to counteract the advance of OA. Some proteins could be potential therapeutic agents in the OA treatment, such as CUR and CTS, that reduce OA progression and chondrocyte apoptosis by epigenetic regulation of miRNAs expression, and especially for CUR upregulating miR-143 and miR-124 [36] or by downregulating miR-574-5p in the case of CTS [28].

In other cases, proteins should be studied as potential therapeutic agents that improve the activity of non-coding RNAs or enzymes that interfere with the methylation level of miRNAs. Examples may be SNHG9 and CircFADS2, which downregulate miR-34a and miR-19-5p, so inhibiting chondrocyte apoptosis [29,30], FTO and DNMT3B that, through methylation of miR-515-5p and miR-34a, respectively, alleviate OA, reduce chondrocyte apoptosis, ECM degradation and inflammatory response [32,35].

Other pharmacological agents should reduce the activity of Circ_HECW2 that increases chondrocyte apoptosis by decreasing miR-63 expression [38] and the activity of EZH2, inhibiting miR-138 expression and inducing cartilage degeneration [34]. Moreover, observing the evolution of the oncology therapy based on DNA methylation inhibitors, it is possible to hypothesize that the data obtained by their use can be a new frontier for future OA treatments. Future studies are needed to verify this hypothesis.

These pieces of evidence enable us to identify new targets for personalized therapeutic approaches for OA care. Some aspects remain to be elucidated that could provide a turning point, enabling (i) a deeper understanding of the different possible involvement of epigenetic protein machines in miRNA regulation during OA disease progression; (ii) the identification and characterization of possible elective therapeutic approaches that, by modulating miRNA methylation state, should be able to limit the inflammation correlated to the disease; and (iii) the role of other different epigenetic modifications in miRNA expression during OA disease.

## Figures and Tables

**Table 1:** The 12 studies grouped based on miRNA upregulation or deregulation in OA tissues and cells.

Aim	In Vitro Model	Analyses	Results	Conclusions in OA	Ref.
Evaluation of CTS on **miR-574-5p** methylation in chondrocyte apoptosis during OA	Chondrocytes from mice (4 days) treated with IL1β. (1) Cells+CTS; (2) Cells+CTS+5-aza-CdR; (3) Cells+CTS and transfected with: miR-574-5p mimic; oe-YAF2	RT-qPCR (miR-574-5p and *Yaf*2); WB (Bax, Bcl-2); Flow cytometry (cell apoptosis); CCK-8 assay (cell proliferation); Dual-luciferase reporter assay (relationship between miR-574-5p and *Yaf2*); MSP (miR-574-5p methylation)	Cells: ↑ miR-574-5p, Bax, apoptosis; ↓ *Yaf2*, Bcl-2, cell proliferation, ↓miR-574-5p promoter methylation. Cells+CTS: ↓ miR-574-5p, Bax, apoptosis; ↑ *Yaf2*, Bcl-2, cell proliferation, **miR-574-5p promoter methylation.** Cells+CTS+5-aza-CdR: ↓ miR-574-5p promoter methylation. Cells+CTS+miR-574-5p mimic: ↑ miR-574-5p, Bax, apoptosis; ↓ *Yaf2*, Bcl2, cell proliferation. Cells+CTS+miR-574-5p mimic +oe-YAF2: ↑ *Yaf2*, cell proliferation; ↓ Bax, cell apoptosis	- miR-574-5p upregulated. - Bax is upregulated, while *Yaf2* and Bcl-2 are downregulated. - Negative correlation between miR-574-5p and *Yaf2* and Bcl-2. - Positive correlation between miR-574-5p and Bax. - **CTS reduces miR-574-5p and increases miR-574-5p promoter methylation**. - CTS could affect chondrocyte proliferation and apoptosis by regulating the expression of miR-574-5p and then interfering with *Yaf2*.	Yue 2021 [28]
Evaluation of the interaction between SNHG9 and **miR-34a** methylation in chondrocyte apoptosis during OA	(1)SF from OA pz (n = 60). (2) Chondrocytes from OA (n = 60) or healthy donors (n = 60) and transfected with: oe-SNHG9; miR-34a mimic	RT-qPCR (*SNHG9*, miR-34a); ELISA (*C*ASPASE*-3*); Flow cytometry (cell apoptosis); MSP (miR-34a methylation)	OA SF: ↓ *SNHG9*; ↑ miR-34a, CASPASE-3, cell apoptosis. OA cells+oe-SNHG9: ↓ miR-34a, apoptosis, CASPASE-3; ↑ **miR-34a methylation**. OA cells+miR-34a mimic: ↑ apoptosis, CASPASE-*3*. Healthy cells+oe-SNHG9: no changes in miR-34a, miR-34a methylation, apoptosis, CASPASE-3. Healthy cells+miR-34a mimic: no changes in *SNHG9*, apoptosis, CASPASE-*3*.	- miR-34a upregulated. - Negative correlation between *SNHG9* and miR-34a. - **Overexpression of SNHG9 decreases miR-34a through methylation**. - Overexpression of SNHG9 decreases chondrocyte apoptosis through miR-34a	Zhang 2020 [29]
Evaluation of the interaction between circFADS2 and **miR-195-5p** methylation in chondrocyte apoptosis during OA	(1) SF from OA pz (n = 63); (2) Purchased human OA chondrocytes transfected with: oe-circFADS2; miR-195-5p mimic	RT-qPCR (circFADS2, miR-195-5p); Flow cytometry (cell apoptosis); MSP (miR-195-5p methylation).	OA SF: ↓ circFADS2; ↑ miR-195-5p. Cells+oe-circFADS2: ↓ miR-195-5p, apoptosis; **↑ miR-195-5p methylation**. Cells+miR-195-5p mimic: ↑ apoptosis; no changes in circFADS2 expression	- miR-195-5p upregulated. - Negative correlation between CircFADS2 and miR-195-5p. - **Overexpression of circFADS2 decreases miR-195-5p expression through methylation**. - Overexpression of circFADS2 decrases chondrocytes apoptosis through miR-195-5p	Zhang 2021 [30]
Evaluation of the relationship between DNMT3B and **miR-29b** methylation in cell apoptosis, ECM synthesis and inflammation in OA	(1) Cartilage or chondrocytes from OA pz (n = 46) treated with IL1β and transfected with: sh-DNMT3B; sh-CDK4; oe-DNMT3B; oe-PTHLH; miR-29b mimic; miR-29b inhibitor	RT-qPCR (*DNMT3B*, miR-29b, *PTHLH*, *CDK4*, *RUNX2*); WB (DNMT3B, PTHLH, CDK4, Ki67, Aggrecan, MMP3, MMP13); MTT (cell viability); Flow cytometry (cell apoptosis); IHF (Ki67, Aggrecan); MSP (miR-29b promoter methylation); Dual-luciferase reporter gene assay (relationship between miR-29b and PTHLH).	OA cartilage: ↓ *DNMT3B*, *PTHLH*, *CDK4*, **miR-29b promoter methylation**; ↑ miR-29b. Cells: ↓ **miR-29b methylation**; ↑ miR-29b expression. Cells+oe-DNMT3B: ↑ **miR-29b methylation**, *PTHLH*, *CDK4*, ubiquitinated RUNX2, cell viability, Ki67, Aggrecan; ↓ miR-29b expression, apoptosis, MMP3, MMP13. Cells+miR-29b mimic: ↓ luciferase activity of *PTHLH*. Cells+miR-29b inhibitor: ↑ *PTHLH*. Cells+sh-DNMT3B: ↓ PTHLH. Cells+oe-PTHLH: ↑ CDK4, cell viability, Ki67 and Aggrecan; ↓ apoptosis, MMP3 and MMP13. Cells+oe-PTHLH+sh-CDK4: ↓ cell viability, Ki67, Aggrecan; ↑ apoptosis, MMP3 and MMP13	- miR-29b upregulated. - Negative correlation between *DNMT3B* and miR-29b. - **Overexpression of *DNMT3B* decreases miR-29b through methylation of the miR-29b promoter**. - *DNMT3B* increases *PTHLH* expression by inhibiting miR-29b. - PTHLH impedes the apoptosis of OA chondrocytes by elevating CDK4	Dou 2021 [31]
Evaluation of the role of **miR-34a** epigenetic mechanism in the degradation of ECM, cell viability and inflammatory response in OA	(1) Cartilage or chondrocytes from OA pz (n = 55) transfected or not with: miR-34a inhibitor; sh-DNMT3B; sh-MCL1.	RT-qPCR (miR-34a, *DNMT3B*, *MCL1*, *MMP3*, *MMP13*, *COL2A1*, *iNOS*, *COX2*, *TNFα*, *IL6*, *PG*); In situ hybridization (miR-34a expression in tissues); CCK-8 (cell viability); Flow cytometry (Cell apoptosis); WB (PI3K/AKT); MSP (miR-34a methylation); Dual-luciferase reporter gene assay (relationship between MCL1 and miR-34a).	OA cartilage and cells: ↑ miR-34a, *MMP3*, *MMP13*, *iNOS*, *COX2*, *TNFα*, *IL6*; *↓ COL2A1*, *MCL1*, *DNMT3B*, **miR-34a methylation**. Cells+sh-DNMT3B: ↑ miR-34a; ↓ MCL1, miR-34a methylation. Cells+miR-34a inhibitor: ↓ *MMP3*, *MMP13*, apoptosis, *iNOS*, *COX2*, *TNFα*, *IL6*; ↑ cell viability, *COL2A1*, *PG*, PI3K/AKT pathway activity, MCL1 luciferase activity. Cells+sh-DNMT3B+sh-MCL1: ↓ cell viability, PG, PI3K/AKT pathway activity; ↑ apoptosis, *iNOS*, *COX2*, *TNFα*, *IL6*.	- miR-34a upregulated. - Positive correlation between miR-34a and *MMP3*, *MMP13*, *iNOS*, *COX2*, *TNFα* and *IL6*. - Negative correlation between miR-34a and *COL2A1*, MCL1 and *DNMT3B*. - ***DNMT3B* suppresses miR-34 through an epigenetic mechanism**. - miR-34a targets and inhibits MCL1 expression. - *DNMT3B*/miR-34a/*MCL1* axis regulates chondrocyte viability. - *DNMT3B*/miR-34a/*MCL1* axis regulates ECM degradation. - *DNMT3B*/miR-34a/*MCL1* axis regulates the inflammatory response. - *DNMT3B*/miR-34a/*MCL1* axis affects chondrocyte viability and OA progression via the PI3K/AKT pathway.	Xiong 2021 [32]
Evaluation of the **miR-128a** methylation effects on chondrocyte survival and articular cartilage anabolism during OA	(1) Cartilage from OA pz (n = 28). 2) 293T cells treated with IL1β transfected with: oe-miR-128; Sh-miR-128. (3) Chondrocytes from 7 days old rats and transfected with: oe-EZH2; EZH2 Rnai; (4) Chondrocytes from 7 days old rats, cultured in micromasses and transfected with: oe-miR-128a; sh-miR-128a	TUNEL staining and flow cytometry (Cell apoptosis); qRT-PCR (*ATG4*, *ATG12*, *p62*, *BECLIN*, *COL2A1*, *AGGRECAN*, *SOX9*, *IL1β*, *CXCL9*); WB (LC3, BAX, BCL2,CASPASE-3, EZH2, H3K27me1, H3K27me2, H3K27me3); Dual-Luciferase reporter assay (relationship between ATG12 and miR-128a); ChIP (EZH2 and H3K27me3 binding on miR-138 promoter).	OA cartilage and cells: ↑ miR-128a expression; ↓ *ATG*12 expression, LC3, H3K27. Cells+oe-miR-128: ↓ 3′-UTR luciferase activity of *ATG*12, *ATG*12 expression, LC3 concentration. Cells+sh-miR-128: ↑ 3′-UTR luciferase activity of *ATG*12, *ATG*12 expression, LC3 concentration. Cells+oe-EZH2: ↑ **methylated** **H3K27 and H3K27me2 enrichment in the proximal region of the miR-128a promoter**, *ATG*12 and LC3; ↓ miR-128a expression. Cells+EZH2 Rnai: ↓ **methylated H3K27, H3K27me2 enrichment to the miR-128a promoter region,** *ATG*12, LC3; ↑ miR-128a expression. Cell micromasses+oe-miR-128a: ↑ BAX, BCL2, and cleaved caspase-3, annexin-V, apoptosis; ↓ Alcian blue staining, *SOX9*, *COL2A1*, and *AGGRECAN*. Cell micromasses+sh-miR-128a: ↓ Bax, Bcl-2, cleaved caspase-3, apoptosis; ↑*SOX9*, *COL2A1*, and *AGGRECAN*.	- miR-128a upregulated. - Negative correlation between miR-128a and *ATG*12, EZH2 and H3K27. - **Overexpression of EZH2 decreases miR-128a through methylation of histone H3K27**. - miR-128a binds directly to the 3′-UTR of *ATG*12. - miR-128a reduces survival and cartilage formation capacity of chondrocytes	Lian 2018 [33]
Evaluation of the biological effects of **miR-23a-3p** methylation in ECM synthesis in OA	(1) Cartilage from OA pz (n = 10). (2) Purchased SW1353 cells transfected with: miR-23a-3p mimic; miR-23a-3p inhibitor; sh-SMAD3	qRT-PCR (*SMAD3*, *COL2A1*, *AGGRECAN*, miR-23a-3p); WB (SMAD3, COL2A1, AGGRECAN); MSP (miR-23a-3p promoter methylation); Dual-luciferase reporter assay (relationship between SMAD3 and miR-23a-3p)	OA cartilage: ↑ miR-23a-3p; ↓ SMAD3, **miR-23a-3p promoter methylation.** Cells+miR-23a-3p mimics: ↓ luciferase activity of *SMAD3*,, *COL2A1*, *AGGRECAN*. Cells+miR-23a-3p inhibitor: ↑ SMAD3, COL2A1, AGGRECAN. Cells+sh-SMAD3: ↓ SMAD3. Cells+ miR-23a-3p inhibitor+sh-SMAD3: ↓ SMAD3, COL2A1, AGGRECAN.	- miR-23a-3p upregulated. - Negative correlation between miR-23a-3p and *SMAD3*. - **Hypomethylation of the miR-23a-3p promoter explains the increased expression of miR-23a-3p**. - *SMAD3* is a target of miR-23a-3p. - miR-23a-3p overexpression suppresses ECM synthesis. - *SMAD3* is essential in the miR-23a-3p-induced downregulation of *AGGRECAN* and *COL2A1*.	Kang 2016 [2]
Evaluation of the molecular mechanism of EZH2/**miR-138**/SDC1 and epigenetic regulation in cell apoptosis and inflammation in OA	(1) Cartilage and chondrocytes from OA pz (n = 25). (2) Chondrocytes from healthy donors treated with IL1β and transfected or not with: sh-EZH2; miR-138 mimic; oe-EZH2; oe-EZH2	RT-qPCR (*EZH2*, miR-138, *SDC1*, *MMP13*, *ADAMTS4*, *ADAMTS5*); WB (EZH2, SDC1); Flow Cytometry (cell apoptosis); Dual-luciferase reporter assay (relationship between SDC1 and miR-138); ChIP (EZH2 and H3K27me3 binding on miR-138 promoter).	OA cartilage and chondrocytes: ↑ *EZH2*, *SDC1*; *↓* miR-138. Cells from healthy donors+IL1β: ↑ *EZH2*, *SDC1*, apoptosis, *MMP13*, *ADAMTS4*, *ADAMTS5*, **miR-138 promoter histone methylation**; ↓ miR-138. Cells+sh-EZH2: ↓ *EZH2*, apoptosis, *MMP13*, *ADAMTS-4*, *ADAMTS-5*, **miR-138 promoter histone methylation**; ↑ miR-138. Cells+oe-EZH2: ↑ EZH2, *SDC1*, apoptosis, *MMP13*, *ADAMTS4*, *ADAMTS5*, **miR-138 promoter histone methylation; ↓ miR-138.** Cells+miR-138 mimic: ↓ *SDC1*, SDC1 luciferase activity. Cells+oe-EZH2+mir-138 mimic: ↑ miR-138; ↓ *SDC1*, apoptosis, *MMP13*, *ADAMTS4*, *ADAMTS5.*	- miR-138 deregulated. - EZH2 is upregulated and promotes OA progression. - Negative correlation between *EZH2* and *SDC1* with miR-138. - **EZH2 inhibits miR-138 expression by increasing histone methylation at its promoter**. - *SDC1* is a target of miR-138. - EZH2 induces cartilage catabolism-related factors by regulating miR-138*/SDC1* signalling	Wang 2021 [34]
Evaluation of the relationship between FTO and **miR-515-5p** in m6A-dependent manner on cell apoptosis and inflammation in OA	(1) Purchased human OA C28/I2 chondrocytes treated with LPS transfected or not with: oe-FTO; sh-FTO; miR-515-5p inhibitor; oe-TLR4	CCK-8 (cell viability); Flow cytometry (cell apoptosis); WB (BCL-2, BAX, CLEAVED-CASPASE-3, FTO, TLR4, MYD88, P/T-P65, P/T-IΚBα); ELISA (IL-6, IL-1β, and TNF-α); m6A RNA methylation level; RT-qPCR (*COX-2*, *iNOS*, *FTO*, miR-515-5p and pri-miR-515-5p); MeRIP (m6A RNA immunoprecipitation); Dual-luciferase reporter assay (relationship between pri-miR-515-5p and DGCR8).	Cells: ↓ cell viability, BCL-2, miR-515-5p expression, FTO; ↑ apoptosis, BAX, CLEAVED-CASPASE-3, COX-2 and iNOS expression, IL-6, IL-1β, and TNF-α, COX-2, pri-miR-515-5p **m6A methylation level**, TLR4, MyD88, p/t-p65, and p/t-IκBα. Cells+oe-FTO: ↓ cell apotosis, BAX, CLEAVED-CASPASE-3, IL-6, IL-1β, and TNF-α, COX-2, iNOS, pri-miR-515-5p m6A methylation level, TLR4, MYD88, P/T-P65, AND P/T-IΚBα; ↑ cell viability, BCL-2, miR-515-5p expression, pri-miR-515-5p binding level to DGCR8. Cells+sh-FTO: ↓ FTO expression, miR-515-5p expression; ↑ **pri-miR-515-5p m6A methylation level.** Cells+oe-FTO+miR-515-5p inhibitor: ↓ miR-515-5p, cell viability, Bcl-2; ↑ apoptosis, Bax, cleaved-caspase-3, IL-6, IL-1β, TNF-α, COX-2, iNOS, TLR4. Cells+oe-FTO+oe-TLR4: ↑ MyD88, p/t-p65, and p/t-IκBα	- miR-515-5p deregulated. - FTO reduced. - **FTO interacts with DGCR8 and modulates the pri-miR-515-5p processing in an m6A dependent manner**. - FTO alleviates OA injury by regulating miR-515-5p. - miR-515-5p inhibits the MyD88/NF-κB pathway by targeting TLR4. - FTO might inhibit TLR4 levels by targeting miR-515-5p.	Cai 2023 [35]
Evaluation of the **miR-143** methylation underlying the role of CUR in OA treatment	(1) Purchased primary chondrocytes treated with IL1β with or without: BMSC-Exos; BMSC-Exos+CUR. (2) Primary chondrocytes+CUR. (3)Primary chondrocytes transfected with: miR-143 mimics; miR-124 mimics	qRT-PCR (miR-124, miR-143, *Rock1*, *NF-kB*); CCK-8 (cell proliferation); Annexin V assay (Cell apoptosis); WB (Rock1, Tlr9, Nf-kB); Bisulphite sequencing; Dual-luciferase reporter assay (relationship between NF-kB and miR-124 or ROCK1 and miR-143)	Cells: ↓ cell viability, miR-124, miR-143; ↑ cell apoptosis, *Rock1*, *Tlr9*, *Nf-kB.* Cells+BMSC-Exos and cells+BMSC-Exos+CUR: ↑ cell viability, miR-124, miR-143; ↓ cell apoptosis, Rock1, Tlr9, Nf-kB. Cells+CUR: ↓ **methylation of miR-143 and miR-124 promoters;** ↑ miR-143 and miR-124. Cells+miR-143 mimics: ↓ 3′-UTR luciferase activity of *Nf-kB*. Cells+miR-124 mimics: ↓ 3′-UTR luciferase activity of *Rock1*.	- miR-124 and miR-143 deregulated. - Exosomes derived from CUR treated MSCs maintain the viability of chondrocytes and protects chondrocytes against IL-1β-induced apoptosis. - Exosomes derived from CUR treated MSCs restore the expression of miRNAs and genes related to OA. - **CUR up-regulates *miR-143* and *miR-124* expression by reducing the DNA methylation of their promoters.** - miR-143 and miR-124 act by inhibiting *Rock1* and *Nf-kB* gene target.	Qiu 2020 [36]
Evaluation of the role of **miR-140-5p** and **miR-146a** methylation in OA	(1)Chondrocytes and synoviocytes from OA pz (n = 20) treated or not with 5-AzadC and trasfected or not with: sh-SMAD3; miR-140-5p inhibitor; sh-NF-kB; miR-146a inhibitor.	RT-qPCR (miR-140, miR-146a, *MMP13*, *ADAMTS5*, *TRAF-6*, *IRAK-1*, *IL6*, *IL1β*, *TNFA*); MSP (specific regions of miR-140 and miR-146a promoter methylation); Bisulfite sequencing (miR-146a promoter methylation); ChIP (NF-kB or SMAD3 binding on miR-146a promoter).	Chondrocytes: ↓ miR-140-5p binding affinity of SMAD3; **↑ miR-140 regulatory region methylation.** Synoviocytes: ↓ **miR-146a, NF-kB binding on miR-146a promoter; ↑ miR-146a promoter methylation.** Chondrocytes+5-AzadC: ↓ **miR-140 regulatory region methylation,** *MMP13*, *ADAMTS5*; ↑ *miR-140-5p*. Synoviocytes+5-AzadC: ↓ miR-**146a promoter methylation,** *IRAK-1*, *IL1β*, *IL6*; ↑ miR-146a. Chondrocytes+5-AzadC+sh-SMAD3: ↓ miR-140-5p. Chondrocytes+5-AzadC+miR-140-5p inhibitor: ↑ *MMP13*, *ADAMTS5*. Synoviocytes+5-AzadC+sh-NF-kB: ↓ miR-146a. Synoviocytes+5-AzadC+miR-146a inhibitor: ↑ *IRAK-1*, *IL1β*, *IL6*. Synoviocytes+miR-146a: ↓ *IRAK-1*, *IL1β*, *IL6.*	- miR-140-5p and miR-146a deregulated. - **miR-140 regulatory region methylation in chondrocytes**. - Negative correlation between miR-140-5p expression and miR-140 regulatory region methylation in chondrocytes. - **miR-146a promoter methylation in synoviocytes**. - Negative correlation between miR-146a expression and miR-146a promoter methylation in synoviocytes. - Methylation impairs SMAD3 binding affinity on miR-140 regulatory region. - MiR-146a promoter methylation impairs the binding affinity of NF-kB. - Methylation mediates downregulation of miR-140-5p on *MMP-13* and *ADAMTS5* expression levels. - Methylation mediates miR-146a on the expression of inflammatory factors involved in OA	Papathanasiou 2019 [37]
Evaluation of the role of **Circ_HECW2** and **miR-93** methylation in cell apoptosis in OA	(1) SF from OA pz (n = 64). (2) Purchased human OA chondrocytes transfected with: oe-Circ_HECW2; miR-93 mimic	RT-qPCR (Circ_HECW2, miR-93); Flow cytometry (cell apoptosis); MSP (miR-93 methylation)	OA SF and cells: ↑ Circ_HECW2; ↓ miR-93. Cells+oe-Circ_HECW2: ↓ miR-93; ↑ **miR-93 methylation**, apoptosis. Cells+miR-93 mimic: ↓ apoptosis; no changes in Circ_HECW2	- miR-93 deregulated. - Circ_HECW2 upregulated. - Negative correlation between Circ_HECW2 and miR-93. - **Circ_HECW2 overexpression decreases miR-93 expression through methylation**. - Circ_HECW2 overexpression increases chondrocyte apoptosis via miR-93	Zuo 2021 [38]

Abbreviations: 5-aza-CdR/5-AzdC = 5-AZA-2’-deoxycytidine; AC008 = AC008440.5; ADAMTS4/5 = ADAM Metallopeptidase With Thrombospondin Type 1 Motif 4/5; ANKH = ANK ilosis H omolog; AQP1 = Aquaporin 1; Atg4/12 = Autophagy-regulating protease 4/12; BAX = Bcl-2-associated X protein; Bcl-2 = B-cell lymphoma-2; CCK-8 = Cell Counting Kit-8; CDK4 = Cyclin Dependent Kinase 4; ChIP = Chromatin Immunoprecipitation; COL2A1 = Collagen II; COX2 = Cyclooxygenase 2; CTS = Cryptotanshinone; CUR = Curcumin; CXCL9 = Chemokine (C-X-C motif) ligand 9; DGCR8 = DiGeorge critical region-8; DNMT3B = DNA Methyltransferase 3 Beta; ECM = Extracellular matrix; Exos = exosomes; EZH2 = Enhancer Of Zeste 2 Polycomb Repressive Complex 2 Subunit; FTO = FTO Alpha-Ketoglutarate Dependent Dioxygenase; H3K27me = Recombinant Histone H3 monomethyl Lys27; IHF = immunofluorescence; IkBα = nuclear factor of kappa light polypeptide gene enhancer in B-cells inhibitor, alpha; IL1β/6 = Interleukin 1 beta/6; iNOS = inducible Nitric oxide synthase; IRAK-1 = interleukin-1 receptor-associated kinase 1; LC3 = Microtubule-associated protein 1A/1B-light chain 3; LPS = Lipopolysaccharides; M6A = N6-methyladenosine; MCL1 = myeloid cell leukemia-1; MMP13/3 = Metalloproteinases 13/3; MSP = Methylation-specific PCR; MSPCR = Methylation-specific PCR; MTT = (3-[4,5-dimethylthiazol-2-yl]-2,5 diphenyl tetrazolium bromide); MyD88 = Myeloid differentiation primary response protein 88; NF-kB = nuclear factor kappa-light-chain-enhancer of activated B cells; OA = osteoarthritis; Oe- = overexpression plasmid; PARP1 = Poly [ADP-ribose] polymerase 1; PG = Prostaglandin; PI3K/AKT = Phosphoinositide 3-kinase/serine-threonine-protein kinase; PTHLH = Parathyroid Hormone Like Hormone; Pz = patients; RIP = RNA Immunoprecipitation Chip; Rnai = RNA interference; ROCK1 = rho-associated, coiled-coil-containing protein kinase 1; RT-qPCR = Quantitative reverse transcription PCR; RUNX2 = Runt-related transcription factor 2; SDC1 = Syndecan 1; SF = synovial fluid; Sh- = Gene Silencers; SMAD3 = small mother against decapentaplegic; SNHG9 = Small nucleolar RNA host gene 9; SOX9 = SRY-box transcription factor 9; TLR4 = Toll Like Receptor 4; TNFα = Tumor Necrosis Factor α; TRAF-6 = TNF Receptor Associated Factor 6; WB = Western Blot; YAF2 = YY1 Associated Factor.
